# Optimizing Row Ratio Configurations for Enhanced Productivity and Resource-Use Efficiency in Maize–Alfalfa Intercropping

**DOI:** 10.3390/plants14243846

**Published:** 2025-12-17

**Authors:** Zeqiang Shao, Shiqiang Hu, Chunying Fan, Ziqing Meng, Xishuai Yan, Wenzhao Ji, Zhihao Zhang, Huimin Ma, Jamal Nasar, Harun Gitari

**Affiliations:** 1 College of Resource and Environment Engineering, Jilin University of Chemical Technology, Jilin 132022, China; hushiqiang1232025@163.com (S.H.); mengziqing1232025@126.com (Z.M.); 15027635853@163.com (X.Y.); 17735470525@163.com (W.J.); r1134860334@163.com (Z.Z.); 2 Zhejiang Zhongdi Jingtu Technology Co., Ltd., Zhuji 311800, China; 15944263582@163.com; 3 Faculty of Agronomy, Jilin Agricultural University, Changchun 130118, China; 4 Institute of Rice Industry Technology Research, College of Agriculture, Guizhou University, Guiyang 550025, China; jamalnasar554@gmail.com; 5 Department of Agricultural Science and Technology, School of Agriculture and Environmental Sciences, Kenyatta University, P.O. Box 43844-00100, Nairobi 00100, Kenya; hgitari@gmail.com

**Keywords:** competitive interaction, crop yield, forage production, intercropping, resource use efficiency

## Abstract

Maize–alfalfa intercropping is practiced in Northeast China to improve land productivity and forage production. However, competition between the two crops can reduce system performance, which calls for an emphasis on optimal row ratio. Hence, the current study evaluated the effects of diverse maize–alfalfa row ratio configurations (1:1, 2:1, 2:2, 3:1, 3:2, and 3:3) on resource-use efficiency, physiological traits, and yield performance. It was noted that the mono-cropping system had higher physiological and agronomic values for both crops. With regard to the intercropping configuration, the 2:2 steadily outperformed all other intercropping row ratios. Whereas alfalfa grew tallest in 2:2, maize plant height peaked under the 3:1. Photosynthetic rate and chlorophyll content were highest under 2:2, for both crops. The yield results indicated that alfalfa achieved maximum forage and biomass, whereas maize performed best under a 3:1 configuration. Outstandingly, under the 2:2 ratio, the cumulative system yield exceeded alfalfa mono-cropping by 55% and maize mono-cropping by 56–57%. There was superior complementarity and land-use advantage under 2:2, as indicated by the highest resource-use indicators of LER (land equivalent ratio), LEC (land equivalent coefficient), SPI (system productivity index), and K (crowding index). Competitive Indices showed that competition was more balanced under 2:2, with maize dominating in systems with higher maize proportions. Overall, the 2:2 row ratio provided the best balance of reduced competition and enhanced complementarity, offering a more efficient and sustainable maize-alfalfa intercropping strategy.

## 1. Introduction

Northeastern China is a major agro-pastoral region that plays a vital role in China’s agriculture and livestock production [1,2,3]. However, the region faces persistent challenges such as soil erosion, low vegetation cover, and insufficient resource use, which collectively undermine ecosystem stability and productivity. Improving resource use efficiency while maintaining stable yield requires a sustainable cropping system that maximizes production and utilizes resources more efficiently [4,5,6]. In this region, grain-forage mixed systems, particularly those involving maize–alfalfa, have shown strong potential to improve ecological sustainability while increasing overall production.

Intercropping, a long-standing agricultural practice, entails the cultivation of at least two crops on the same land, and it is widely used, especially in China, due to its ecological and agronomic benefits [7,8,9]. In comparison with mono-cropping, intercropping heightens the exploitation of nutrients, light, and water, land by utilizing spatial and temporal differences between component crops [10,11,12]. Yield advantages are often driven by complementarity and facilitation: complementarity reduces competition by allowing crops to access resources differently, while facilitation occurs when one crop improves the growth environment of the other [13,14]. These interactions help sustain greater system productivity, particularly through improved light interception, nutrient acquisition, and soil moisture use [8]. However, imbalanced resource demand can intensify interspecific competition, limiting the performance of one or both crops [7,8,10].

Several management strategies, including relay intercropping, strip intercropping, and altered row arrangements, have been developed to reduce competitive pressure [8,15,16]. Amongst these, the spatial proportion of component crops plays a central role, as it determines both the distribution of resources and the intensity of complementarity or competition [17,18]. Unbalanced row proportions result in shading of the recessive crop by the dominant one, where the latter outcompetes the former in utilization of resources [7,19,20]. Contrariwise, balanced spatial arrangements improve system performance by reducing competition and enhancing resource partitioning [7,21]. Previous studies have revealed that cereal grain yield was boosted by increasing cereal row proportions, although with suppressed legume biomass, while with increased legume rows, there was improved forage yield and nitrogen fixation at the expense of cereal yield [18,22]. Consequently, ascertaining an ideal row configuration is vital for attaining synergistic efficient resource use and interactions [23,24].

In northeast China, alfalfa is a key forage legume valued for its high-protein content and soil-improving properties, while maize remains a dominant crop used for grain and forage [3,6,25]. Although numerous studies have evaluated their combined cultivation, the specific influence of different spatial proportions on resource use efficiency, physiological responses, system productivity, and competitive dynamics remains insufficiently understood [2,3,4].

Therefore, this study aimed to identify the optimal spatial proportion for maize–alfalfa mixed cropping that minimizes competition and enhances complementarity, and improves system productivity. We assessed how diverse row arrangements affect maize grain yield, physiological traits, and alfalfa forage yield. The innovation of this study lies in its integrated assessment of physiological responses, competitive interactions, and system-level productivity across multiple spatial configurations, providing a mechanistic understanding of how row proportion shapes resource-use efficiency in the maize-alfalfa intercropping system. The findings provide a basis for designing a more efficient and sustainable intercropping system in northeastern China.

## 2. Results

### 2.1. Plant Height

**Figure 1:**
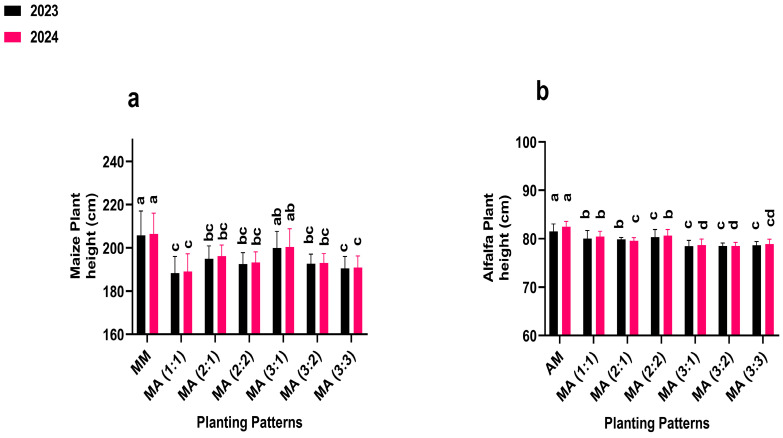
Plant height of maize (**a**) and alfalfa (**b**) under different planting patterns and row ratio configurations. MM: maize mono-cropping, AM: alfalfa mono-cropping, MA (1:1): 1 row of maize intercropped with 1 row of alfalfa, MA (2:1): 2 rows of maize intercropped with 1 row, MA (2:2): 1 rows of maize intercropped with 2 rows of alfalfa, MA (3:1): 3 rows of maize intercropped with 1 row of alfalfa, MA (3:2): 3 rows of maize intercropped with 2 rows of alfalfa, MA (3:3): 3 rows of maize intercropped with 3 rows of alfalfa. Lowercase letters on the bar column represent the significance (*p* ≤ 0.05) difference at the LSD test.

Intercropping row ratio configurations significantly (*p* ≤ 0.05) influenced the plant height of maize and alfalfa (Figure 1). In both 2023 and 2024, maize mono-cropping (control) produced the highest plant height (205.7 and 206.4 cm), exceeding all intercropping treatments. Among the intercropping treatments, the 3:1 row ratio configurations showed the tallest maize plants (199.97 and 200.04 cm in 2023 and 2024), representing 97% of the control. For alfalfa, mono-cropping (control) also achieved the highest plant height (81.5 and 82.4 cm in 2023 and 2024). Within the intercropping system, the 2:2 ratio resulted in the tallest alfalfa plants (80.35 and 80.65 cm in 2013 and 2024), corresponding to 98% of the control.

### 2.2. Chlorophyll and Photosynthetic Rate

**Figure 2:**
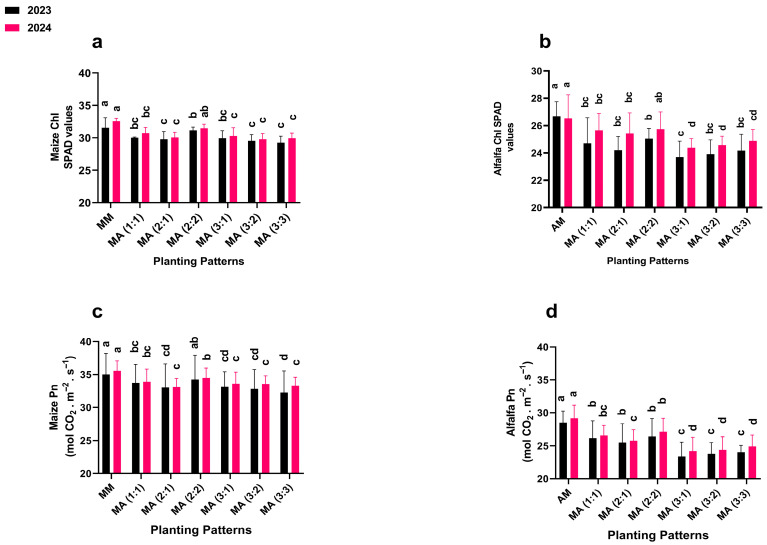
Chlorophyll and photosynthetic rate of maize (**a**,**c**) and alfalfa (**b**,**d**) under different planting patterns and row ratio configurations. MM; maize mono-cropping, AM; alfalfa mono-cropping, MA (1:1); 1 row of maize intercropped with 1 row of alfalfa, MA (2:1); 2 rows of maize intercropped with 1 row, MA (2:2); 1 rows of maize intercropped with 2 rows of alfalfa, MA (3:1); 3 rows of maize intercropped with 1 row of alfalfa, MA (3:2); 3 rows of maize intercropped with 2 rows of alfalfa, MA (3:3); 3 rows of maize intercropped with 3 rows of alfalfa. Lowercase on the bar column represents the significance (*p* ≤ 0.05) difference at the LSD test.

Intercropping row ratio configurations significantly (*p* ≤ 0.05) affected the chlorophyll (Chl) and photosynthetic rate (Pn) of maize and alfalfa (Figure 2). In 2023 and 2024, maize mono-cropping (control) exhibited the highest Chl (31.56 and 32.56) and Pn (35.01 and 35.54), surpassing all intercropping treatments. Among the intercropping treatments, the 2:2 configuration produced the highest maize Chl (31.15 and 31.48) and Pn (34.23 and 34.46) in 2023 and 2024, respectively, representing 98 and 97% of the control, respectively. For alfalfa, mono-cropping (control) also achieved the highest Chl (26.67 and 26.53) and Pn (28.49 and 29.15) in both years. Within the intercropping treatment, the 2:2 showed the maximum alfalfa Chl (25.04 and 25.73) and Pn (26.39 and 27.13), corresponding to 93 and 92% of the control, respectively.

### 2.3. Yield and Yield Attributes

Intercropping row ratio configurations significantly (*p* ≤ 0.05) affected the yield indices of maize and alfalfa (Table 1 and Table 2). In 2023 and 2024, maze mono-cropping (control) produced the highest grain yield (9470.3 and 9947.0 kg ha^−1^), 1000-grain weight (257.42 and 256.44 g), and biomass dry matter (12,085.71 and 12,597.79 kg ha^−1^), exceeding all intercropping treatments. Among the intercropping treatments, the 3:1 row ratio configuration produced the highest maize yield components, with grain yield (8534.01 and 9104.91 kg ha^−1^), 1000-grain weight (248.12 and 248.19 g), and biomass dry (10,848.09 and 11,334.56 kg ha^−1^), representing 91, 96, and 95% of the control, receptively.

For alfalfa, mono-cropping (control) also achieved the maximum forage yield (11,778.64 and 12,300.03 kg ha^−1^) and biomass dry matter (9416.93 and 9807.11 kg ha^−1^) in both years. Among the intercropping treatments, the 2:2 configuration produced maximum alfalfa yield attributes, with forage yield (8366.1 and 8412.2 kg ha^−1^) and biomass dry matter (6868.86 and 7212.97 kg ha^−1^), corresponding to 68 and 75% of the control, respectively.

### 2.4. Cumulative Yield

**Figure 3:**
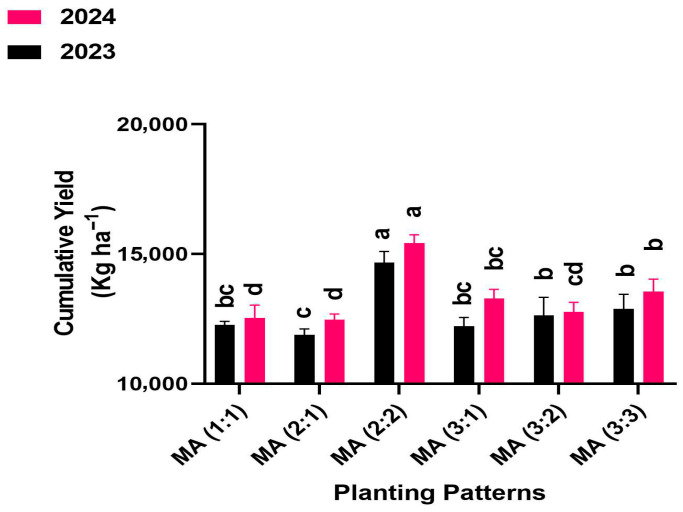
Cumulative yield of maize-alfalfa intercropping under different row ratio configurations. MM: maize mono-cropping, AM: alfalfa mono-cropping, MA (1:1): 1 row of maize intercropped with 1 row of alfalfa, MA (2:1): 2 rows of maize intercropped with 1 row, MA (2:2): 1 rows of maize intercropped with 2 rows of alfalfa, MA (3:1): 3 rows of maize intercropped with 1 row of alfalfa, MA (3:2): 3 rows of maize intercropped with 2 rows of alfalfa, MA (3:3): 3 rows of maize intercropped with 3 rows of alfalfa. Lowercase letters on the bar column represent the significance (*p* ≤ 0.05) difference at the LSD test.

Cumulative yield (sum of maize grain yield and alfalfa biomass) differed significantly among intercropping row ratio configurations. In 2023 and 2024, all intercropping treatments produced the highest cumulative yield compared to mono-cropping (control) maize and alfalfa. Among the intercropping treatments, the 2:2 row configurations recorded the highest cumulative yield, exceeding control and other row configurations (Figure 3). The cumulative yield under 2:2 row configurations was 57% and 55% higher than the control, representing the maximum total productivity among the intercropping treatments.

### 2.5. Resource Use Efficiency Indices

**Figure 4:**
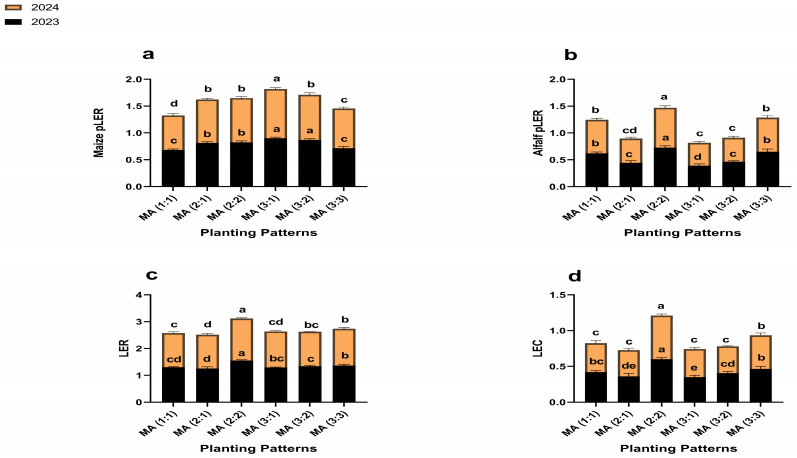
Resource use efficiency indices, maize pLER (**a**), alfalfa pLER (**b**), LER (**c**), and LEC (**d**) under different planting patterns and row ratio configurations. MM: maize mono-cropping, AM: alfalfa mono-cropping, MA (1:1): 1 row of maize intercropped with 1 row of alfalfa, MA (2:1): 2 rows of maize intercropped with 1 row, MA (2:2): 1 rows of maize intercropped with 2 rows of alfalfa, MA (3:1): 3 rows of maize intercropped with 1 row of alfalfa, MA (3:2): 3 rows of maize intercropped with 2 rows of alfalfa, MA (3:3): 3 rows of maize intercropped with 3 rows of alfalfa. Lowercase letters on the bar column represent the significance (*p* ≤ 0.05) difference at the LSD test.

Significant variations were observed in the resource use efficiency indices, such as land equivalent ratio (LER) and land equivalent coefficient (LEC), among the intercropping row ratio configurations (Figure 4). Since LER and LEC are defined only for intercropping treatment, all comparisons were made among intercropping treatments relative to the control baseline (LER = 1, LEC = 0).

In 2023 and 2024, all intercropping treatments recorded LER > 1 and LEC > 0, demonstrating a consistent land use advantage over control. Among the intercropping treatments, the 2:2 produced the highest LER (1.5 in both years) and LEC (0.6 in both years), surpassing all other intercropping treatments.

### 2.6. Productive and Advantageous Indices

**Figure 5:**
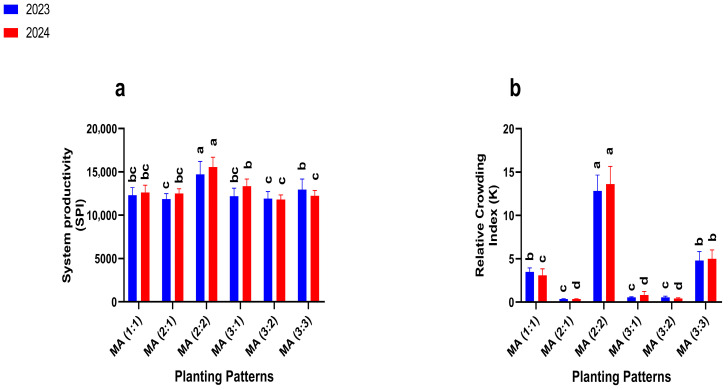
Productive and advantageous indices, system productivity (**a**), and the Relative Crowding Index (**b**) under different planting patterns and row ratio configurations. MM: maize mono-cropping, AM: alfalfa mono-cropping, MA (1:1): 1 row of maize intercropped with 1 row of alfalfa, MA (2:1): 2 rows of maize intercropped with 1 row, MA (2:2): 1 rows of maize intercropped with 2 rows of alfalfa, MA (3:1): 3 rows of maize intercropped with 1 row of alfalfa, MA (3:2): 3 rows of maize intercropped with 2 rows of alfalfa, MA (3:3): 3 rows of maize intercropped with 3 rows of alfalfa. Lowercase letters on the bar column represent the significance (*p* ≤ 0.05) difference at the LSD test.

Intercropping row ratio configurations significantly influenced the System Productivity Index (SPI) and Relative Crowding Index (K) (Figure 5). Since SPI and K were calculated only for intercropping treatments, comparisons were among the intercropping row ratio configurations to identify the most productive and competitive arrangements.

In 2023 and 2024, the SPI values varied markedly among the different intercropping arrangements. Among the intercropping treatments, the 2:2 configuration recorded the highest SPI values (14,724.18 and 15,552.09) and K values (12.82 and 13.63) greater than 1, exceeding all other intercropping arrangements.

### 2.7. Empirical Analysis of Competitive Indices

**Figure 6:**
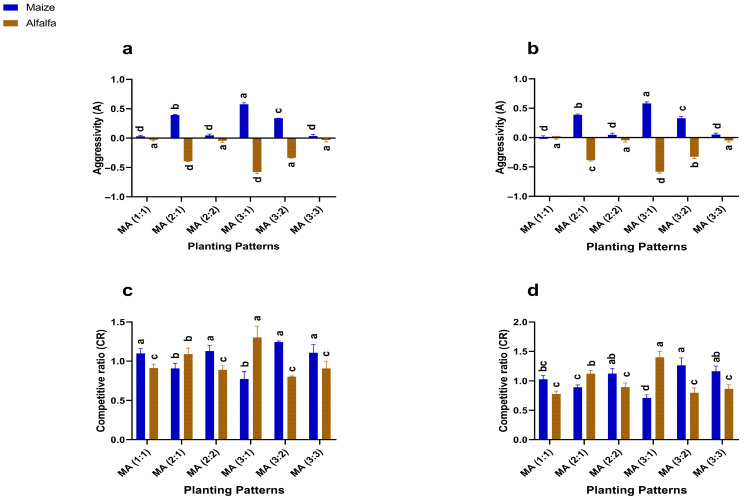
Competitive Indices, aggressivity (**a**,**b**), and competitive ratio (**c**,**d**) under different planting patterns and row ratio configurations in 2023 and 2024, resepctively. MM: maize mono-cropping, AM: alfalfa mono-cropping, MA (1:1): 1 row of maize intercropped with 1 row of alfalfa, MA (2:1): 2 rows of maize intercropped with 1 row, MA (2:2): 1 rows of maize intercropped with 2 rows of alfalfa, MA (3:1): 3 rows of maize intercropped with 1 row of alfalfa, MA (3:2): 3 rows of maize intercropped with 2 rows of alfalfa, MA (3:3): 3 rows of maize intercropped with 3 rows of alfalfa. Lowercase letters on the bar column represent the significance (*p* ≤ 0.05) difference at the LSD test.

Intercropping row ratio configurations significantly affected the Competitive Indices such as aggressivity (A) and competitive ratio (CR) values of maize and alfalfa (Figure 6). Since mono-cropping (control) has no interspecific competition, all Competitive Indices were compared among intercropping treatments relative to the control baseline (A and CR = 0).

In both 2023 and 2024, maize showed the positive A values in all intercropping treatments, indicating dominance over alfalfa compared with the control. The 3:1 configurations recorded the height A values (0.57 and 0.58), reflecting the strongest competitive advantage. In contrast, alfalfa showed negative A values across all intercropping treatments, confirming its lower competitiveness relative to maize. The 1:1 configuration showed the least negative A values (−0.03 and −0.008), indicating reduced suppression compared with the intercropping ratio.

CR values also differed significantly. Compared with control (CR = 0), all intercropping treatments showed CR > 0 for both crops. Maize achieved its highest CR values under a 3:2 configuration (1.24 and 1.26), while alfalfa showed its highest CR values under a 3:1 configuration (1.3 and 1.4), each surpassing all other intercropping treatments.

## 3. Discussion

Intercropping is practiced to improve crop yield and resource use efficiency, such as land, light, and nutrients [26]. However, competition for these resources between intercrops restricts the growth of one or both crops, leading to lower system productivity. Thus, proper management practices are needed to minimize competition and maximize complementarities to build a more stable and productive intercropping system. Spatial row–ratio configuration is one such management and is a critical factor influencing the performance of intercropping systems because it governs how the two species share essential resources such as light, nutrients, water, and rooting space [7,27,28]. In this study, maize–alfalfa intercropping was practiced with different row configurations such as 1:1, 2:1, 2:2, 3:1, 3:2, and 3:3 to identify the optimal spatial proportion that minimizes competition and enhances complementarity, and improves system productivity. Although intercropping produced lower yield and yield indices compared to the mono-cropping maize or alfalfa (control), but produced nearly 90–95% that of the mono-cropping. Nevertheless, among the row ratio configurations, the 2:2 produced the highest (14,676.72 and 15,424.42 kg ha^−1^) cumulative yield in 2023 and 2024, which was 57% higher than mono-cropping maize and 55% over alfalfa mono-cropping. This substantial yield advantage under a 2:2 row configuration might be due to the strength of complementarity, mutual sharing of resources (i.e., light, nutrient, and water) with minimum interspecific competition [7,29]. Moreover, the improved light distribution, reduced shading stress, and a more favorable microenvironment within the canopy under a 2:2 row configuration helped enhance the chlorophyll content (SPAD) and photosynthetic rate (Pn) in both species, resulting in better growth and yield performance at the system level. Similarly, earlier research reports that balanced arrangements optimize total output by enabling more efficient simultaneous use of resources such as nitrogen, light, and water, while dominant configurations favor one crop [30,31]. Some studies emphasize that balanced intercrops improve canopy light penetration, chlorophyll retention, and photosynthetic efficiency, ultimately contributing to superior yield outcomes [8,10,12,32]. The high cumulative yield observed here echoes strong temporal and spatial complementarity between maize and alfalfa under 2:2 spacing, resulting in more complete resource capture and higher overall productivity.

Interestingly, the maize or alfalfa alone achieved its maximum individual yields under specific dominant ratios. For example, the maize crop achieved the highest growth and yield indices, such as plant height, 1000-grain weight, grain yield, and biomass dry matter under a 3:1 configuration. However, alfalfa showed better performance under a 2:2 or 1:1 row configuration. Perhaps, maize requires more light and nutrients for its growth and development, either outcompete alfalfa for available resources or faces less competition from [1]. However, the better growth and yield performances of alfalfa under 2:2 or 1:1 might be due to closer alteration reducing shading by maize and supporting better growth and nitrogen fixation of alfalfa [3]. Previously, it was reported that the heavy shading or intense competition for resources by maize adversely affected alfalfa or soybean under intercropping, resulting in lower growth and yield [1,3,19]. However, with optimal row or strip configuration, this intense competition has lessened, and the system as a whole is superior to mono-cropping [1,3,19].

The present study further demonstrated that intercropping better utilized the available resources as compared to mono-cropping. The resource-use efficiency and system productivity indices suggested that all intercropping row configurations showed more efficient utilization of resources, but the 2:2 configuration had the highest values, confirming an advantage over mono-cropping. For example, the land use indices LER value was highest, 1.5 > 1, and LEC value was 0.6 > 0 under a 2:2 row configuration, more efficiently utilizing the land compared to mono-cropping. Similarly, the system productivity index (SPI) and crowding index (K) values were maximum under a 2:2 row ratio configuration, confirming the intercropping advantageous effect over mono-cropping (control). Collectively, these metrics indicate greater system productivity and superior land-use efficiency under the balanced arrangement [8,10,33,34]. Similarly, earlier studies [17,19] demonstrate that balanced cereal–legume proportions enhance better utilization of the land and other resources, which aligns with the observed performance of the 2:2 system.

The Competitive Indices further confirmed improved coexistence in the balanced configuration and reduced competition in the current study. Under maize-dominant ratios, maize exhibited strong dominance as shown by higher competitive ratio values and positive aggressivity. Nevertheless, under the 2:2 arrangement, competitive ratios approached unity, indicating reduced crop suppression and a more balanced interaction. Such transition to coexistence from dominance mirrors findings from other cereal–legume systems [26,35].

Overall, there was a most favorable balance between competition and complementarity under the 2:2 maize–alfalfa row configuration, resulting in enhanced physiological performance, stabilized yield components, improved system-wide resource-use efficiency maximizing and high cumulative yield. Thus, the 2:2 configuration stands out as a practical and effective strategy for achieving high combined grain and forage production while promoting more efficient and sustainable agricultural systems.

## 4. Materials and Methods

The Jilin University of Chemical Technology, College of Resource and Environmental Engineering, Jilin, China, was the home for the current study. The institute is located at 43°28′1.3308″ N, 123°57′15.2436″ E at an altitude of 248.5 m ASL. The field experiment was conducted here over two consecutive growing seasons (2023 and 2024). The region is characterized by four distinct seasons and semi-arid conditions, which qualify it for a temperate continental monsoon climate. The experimental soil was loamy (alluvial soil) and contained relatively low levels of alkaline nitrogen 80 mg kg^−1^ (alkaline KMnO_4_ method) [36], total nitrogen 1.5 g kg^−1^ (Kjeldahl digestion method) [36], and pH of 6.5 (soil: water = 1:2.5) [37]. In addition, it had available phosphorus of 15 mg kg^−1^ (Olsen P method) [38], available potassium of 115 mg kg^−1^ (Neutral ammonium acetate extraction method) [39], and organic matter of 22 g kg^−1^ (Walkley-Black dichromate oxidation method) [40].

### 4.1. Experimental Design and Layout

**Figure 7:**
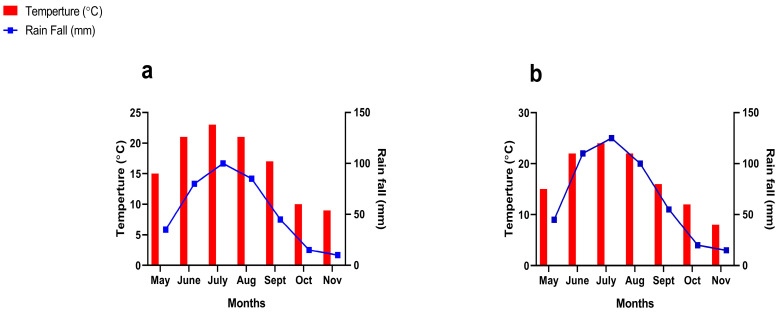
Weather forecast for the experimental during the growth stages in 2023 (**a**) and 2024 (**b**).

Maize (Zhengdan 958) and Alfalfa (Dongmu No. 1) were mono-cropped (i.e., MM: Maize mono-cropping and AM: alfalfa mono-cropping) and intercropped (i.e., MA: maize-alfalfa intercropping) [3] at different row ratio configurations such as 1:1 (1 row of each maize and alfalfa), 2:1 (2 rows of maize with 1 row of alfalfa), 2:2 (2 rows of each maize and alfalfa), 3:1 (3 rows of maize with 1 row of alfalfa), 3:2 (3 rows of maize with 2 rows of alfalfa), and 3:3 (3 rows of each maize and alfalfa) (Supplementary Material). The experimental treatments were arranged in a complete randomized design (CRD) and replicated thrice, with the total plots being 24. The mono-cropping and intercropping were adjusted on different plot sizes (Supplementary Table S1). Before sowing, the experimental soil was well plugged and fertilized with basal NPK application, such as 225 kg N ha^−1^, 120 kg P ha^−1^, and 60 kg K ha^−1^ for maize, and 53 kg N ha^−1^, 135 kg P ha^−1^, and 90 kg K ha^−1^ were applied for alfalfa based on soil test-based fertilizer recommendations and regional agronomic extension guideline [3,6]. For intercropping treatments, fertilizer doses were calculated based on the row proportion of maize and alfalfa in each pattern, and the resulting amounts were uniformly broadcast before sowing. The fertilizer sources used were urea (46% N), triple superphosphate (TSP; phosphorus pentoxide P_2_O_5_ 46% P), and muriate of potash (MOP, KCL; potassium oxide K_2_O 60% K). To maintain consistency and comparability across treatments, the sowing rates were kept identical in mono-cropping and intercropping: maize plants were planted at 60,000 plants ha^−1^, and alfalfa was sown at a seed rate of 15 kg ha^−1^. Maize and alfalfa were sown on 15 May and 10 June in 2023, and 10 May and 15 June in 2024, and harvested on 10 October and 5 November in 2023, and 15 October and 10 November in 2023, respectively. Agricultural field management practices such as irrigation, weeding, pests, and diseases were cautiously monitored and controlled as per standard techniques. The weather report, such as average temperature (in °C) and rainfall (in mm), was collected from the local metallurgical station (30 km away from the experimental site) during the entire season (Figure 7).

### 4.2. Data Collection and Measurements

#### 4.2.1. Physio-Agronomic Indices

##### Plant Height

The representative five plants were randomly selected to measure the plant height. The plant height of maize was measured (in cm) at tasseling, silking, and maturity stages, and that of alfalfa was measured at bud, full bloom, and harvest stages. These measurements were made using a measuring tape, and the averages were calculated accordingly.

##### Yield Components and Biomass Dry Matter

Maize and alfalfa were harvested from representative areas of 5 m^2^ at full maturity. The yield components of maize, such as grain yield and 1000-grain weight (g), and forage yield of alfalfa were measured after harvesting. However, for biomass dry matter, the plant samples were sun-dried, and oven-dried at 105 °C and 80 °C for 0.5 h and 48 h, respectively, to constant weight. The indices were then measured using an electronic scale.

#### 4.2.2. Chlorophyll and Photosynthetic Rate

Chlorophyll and photosynthetic rate (Pn) of maize and alfalfa were measured from the fully expanded uppermost leaf at pre-tasseling, tasseling-silking and milking stages of maize crop, and at early growth, bud and full bloom stages of alfalfa (Equation (1)). In the intercropping system, measurements were taken from both outer and inner rows of each crop species to account for variations in light exposure and canopy position. These measurements were taken between 9:00 and 11:00 a.m. on sunny days using an SPAD-502 Chlorophyll Meter (Minolta, Osaka, Japan) and a Yaxin-1101s photosynthetic analyzer (Beijing Yaxinliyi Science and Technology Ltd., Beijing, China). For Pn, the CO_2_ changes in the plants were monitored for 60 s in a closed-circuit system within a 60 cm L × W photosynthetic chamber [41].

(1) $$Photosynthetic\;Rate[µmol{CO}_{2}\cdot m^{-2}\cdot s^{-1}]=\frac{N_{2}-N_{1}}{0.6\times 0.6}\times 60$$

where N_1_ and N_2_ denote the initial and final concentration of CO_2_ at 60 s, respectively.

### 4.3. Resource Use Efficiency Indices of Maize-Alfalfa Intercropping

#### 4.3.1. Land Equivalent Ratio

The land equivalent ratio (LER), an index used to measure how much land would be required for intercropping to produce the same yield as in mono-cropping, was calculated according to Equation (2) [6].

(2) $$LER={pLER}_{M}+{pLER}_{A}=\frac{Y_{IM}}{Y_{MM}}+\frac{Y_{IA}}{Y_{AM}}$$

where pLER_M_ and pLER_A_ are the partial land equivalent ratios of maize and alfalfa, M is the maize crop, A is the alfalfa crop, Y_IM_ and Y_IA_ are the relative yields of M and A under intercropping, and Y_MM_ and Y_AM_ are the yields of the same species under mono-cropping, respectively.

#### 4.3.2. Land Equivalent Coefficient (LEC)

The land equivalent coefficient (LEC) was used to measure the intercropping efficiency by accounting for the land use proportion and crop yields [42]. It is used to determine whether maize and alfalfa intercropping utilize the land resources more efficiently compared to their mono-cropping. It was computed as a product of the land equivalent ratios (pLER) of maize and alfalfa (Equation (3)).

(3) $$LEC={pLER}_{M}\times {pLER}_{A}$$

### 4.4. Productive and Advantageous Indices of Maize-Alfalfa Intercropping

#### 4.4.1. System Productivity Index (SPI)

System Productivity Index (SPI) is used to determine the total system production to know whether intercropping increases system productivity compared to mono-cropping, and is calculated as shown in Equation (4) [43,44,45].

(4) $$SPI=Y_{IM}+\left(\frac{Y_{MM}}{Y_{AM}}\right)\times Y_{IA}$$

Y_IM_ and Y_IA_ represent the yields of maize and alfalfa under intercropping, while Y_MM_ and Y_AM_ denote their respective yields under mono-cropping.

#### 4.4.2. Relative Crowding Index (K)

The Relative Crowding Index (K) is used to measure the advantageous effect of intercropping by understanding its competition and complementarity between maize and alfalfa, where a K > 1 signposts a benefit of intercropping. It is calculated as shown in Equations (5)–(7).

(5) $$K=K_{IM}\times K_{IA}$$

(6) $$K_{IM}=Y_{IM}\times \frac{Z_{A}}{\left[\left(Y_{MM}-Y_{IM}\right)\times Z_{M}\right]}$$

(7) $$K_{IA}=Y_{IA}\times \frac{Z_{M}}{\left[\left(Y_{AM}-Y_{IA}\right)\times Z_{A}\right]}$$

K_IM_ and K_IA_ epitomize the Relative Crowding Index of maize and alfalfa under intercropping, respectively, while Z_M_ and Z_A_ denote the sowing proportion of maize and alfalfa under intercropping.

### 4.5. Competitive Indices

#### 4.5.1. Aggressivity (A)

Aggressivity (A) is an index used to determine the aggressiveness of one crop to another, to know which shows more dominance during intercropping. It was computed using Equations (8) and (9) [44,46].

(8) $$A=\frac{Y_{IM}}{Y_{MM}\times Z_{M}}-\frac{Y_{IA}}{Y_{AM}\times Z_{A}}$$

where A is the aggressivity of maize and alfalfa. Both crops are deemed to be equally competitive if A = 0. A negative shows alfalfa as the dominant species, whereas a positive value indicates that maize dominates alfalfa.

#### 4.5.2. Competitive Ratio (CR)

The competitive ratio (CR) is used to measure the competitiveness of maize and alfalfa, to determine which crop competes more for resources during intercropping. It was calculated using Equations (9) and (10) [47,48].

(9) $${CR}_{M}=\left(\frac{{pLER}_{M}}{{pLER}_{A}}\right)\times \left(\frac{Z_{A}}{Z_{M}}\right)$$

(10) $${CR}_{A}=\left(\frac{{pLER}_{A}}{{pLER}_{M}}\right)\times \left(\frac{Z_{M}}{Z_{A}}\right)$$

When CR_A_ > 1, it indicates a negative benefit. However, CR_M_ < 1 designates that intercropping offers an optimistic benefit, suggesting that alfalfa can freely be intercropped with maize. If the difference between CR_M_ and CR_A_ is 0, both crops are equally competitive [20,49]. A positive difference shows maize dominates, while a negative value means alfalfa is the dominant species.

### 4.6. Statistical Analyses

Data were analyzed using MS statistix 8.1 after being compiled in MS Excel 2016. Mean comparisons were made using the LSD test at *p* ≤ 0.05. Graphs were created with GraphPad Prism 8.1.

## 5. Conclusions

The current study establishes that maize–alfalfa intercropping performance is highly dependent on row ratio configuration. In terms of grain yield, forage biomass, and overall system productivity, the 2:2 maize–alfalfa row ratio consistently showed superior performance among the tested configurations. Resource-use efficiency indices, such as partial land equivalent ratio (pLER), land equivalent coefficient (LEC), land equivalent ratio (LER), and system productivity index (SPI), indicated that the 2:2 configuration maximized the complementary utilization of water, light, and nutrients. Competition indices, including aggressivity, crowding coefficient, and competitive ratio, established balanced interspecific interactions, with maize and alfalfa excellently coexisting, deprived of strong overpowering of either crop. The results suggest that balanced row ratios optimize complementarity and reduce interspecific competition, making them the most suitable arrangement for achieving high crop yield and efficient resource utilization in maize–alfalfa intercropping systems. These findings provide practical guidance for farmers and agricultural planners in Northeastern China and similar agro-ecological regions, supporting sustainable intensification and improved forage-grain production.

## Figures and Tables

**Table 1:** Yield and yield indices of maize crop under different planting patterns at different row ratio configurations.

Planting Patterns	Grain Yield (Kg ha^−1^)		Biomass Dry Matter (Kg ha^−1^)		1000-Grain Weight (g)	
	2023	2024	2023	2024	2023	2024
MM	9470.3 ± 813.05 a	9947.0 ± 680.19 a	12,086.4 ± 1479.7 a	12,598.3 ± 1176.8 a	257.43 ± 8.9 a	256.45 ± 10.9 a
MA (1:1)	6429.1 ± 363.13 e	6386.2 ± 320.48 e	8564.3 ± 1109.8 d	9545.1 ± 1388.1 d	229.14 ±7.8 c	221.76 ± 7.5 c
MA (2:1)	7691.3 ± 462.36 d	8012.5 ± 294.96 d	9706.1 ± 1257.7 c	10,141.4 ± 1000.3 c	235.96 ±6.3 bc	235.98 ± 7.9 bc
MA (2:2)	7800.9 ± 575.11 cd	8211.4 ± 308.56 c	10,277.2 ± 1331.7 bc	10,738.2 ± 1059.1 bc	236.78 ± 4.7 bc	242.80 ± 15.2 ab
MA (3:1)	8534.0 ± 922.06 b	9104.9 ± 343.56 b	10,848.4 ± 1405.7 b	11,335.1 ± 1117.9 b	248.18 ± 18.6 ab	248.40 ± 15.6 ab
MA (3:2)	8245.8 ± 846.66 bc	8370.8 ± 913.50 c	9935.1 ± 1287.3 c	10,380.3 ± 1023.8 bc	237.39 ± 17.8 bc	237.41 ± 14.9 bc
MA (3:3)	6771.4 ± 317.67 e	7317.2 ± 319.21 e	9706.3 ± 1257.7 c	10,141.2 ± 1000.3 c	233.27 ± 6.1 bc	233.28 ± 9.4 bc
LSD	0.000 ***	0.000 ***	0.000 ***	0.000 ***	0.044 **	0.013 **

The means with different lowercase letters (±SD) are significantly different from each other at the LSD test *p* ≤ 0.05 level of probability. MM: maize mono-cropping, MA (1:1): 1 row of maize intercropped with 1 row of alfalfa, MA (2:1): 2 rows of maize intercropped with 1 row of alfalfa, MA (2:2): 2 rows of maize intercropped with 2 rows of alfalfa, MA (3:1) 3 rows of maize intercropped with 1 row of alfalfa, MA (3:2): 3 rows of maize intercropped with 2 rows of alfalfa, MA (3:3): 3 rows of maize intercropped with 3 rows of alfalfa. LSD; least significant difference, *** (*p* ≤ 0.001), ** (*p* ≤ 0.01).

**Table 2:** Yield and yield indices of alfalfa crop under different planting patterns at different row ratio configurations.

Planting Patterns	Forage Yield (Kg ha^−1^)		Biomass Dry Matter (Kg ha^−1^)	
	2023	2024	2023	2024
MM	11,779.3 ± 558.00 a	12,300.0 ± 359.26 a	9416.9 ± 668.31 a	9807.1 ± 660.40 a
MA (1:1)	8012.0 ± 570.06 b	8377.4 ± 653.80 b	5835.1 ± 409.64 c	6139.6 ± 314.17 c
MA (2:1)	6917.2 ± 895.83 c	6385.5 ± 1312.70 d	4184.5 ± 579.91 de	4450.7 ± 454.34 d
MA (2:2)	8366.1 ± 618.64 b	8412.2 ± 596.88 b	6868.3 ± 455.07 b	7213.1 ± 378.58 b
MA (3:1)	5245.1 ± 281.60 d	5120.9 ± 816.24 d	3687.1 ± 588.45 e	4179.5 ± 285.58 d
MA (3:2)	5655.1 ± 161.97 d	5646.3 ± 228.63 d	4393.3 ± 314.96 d	4391.6 ± 539.29 d
MA (3:3)	6867.4 ± 488.62 c	7180.2 ± 560.40 bc	6111.8 ± 488.39 c	6232.3 ± 340.38 c
LSD	0.000 ***	0.000 ***	0.000 **	0.000 **

The means with different lowercase letters (±SD) are significantly different from each other at the LSD test *p* ≤ 0.05 level of probability. MM: maize mono-cropping, MA (1:1): 1 row of maize intercropped with 1 row of alfalfa, MA (2:1): 2 rows of maize intercropped with 1 row of alfalfa, MA (2:2): 2 rows of maize intercropped with 2 rows of alfalfa, MA (3:1): 3 rows of maize intercropped with 1 row of alfalfa, MA (3:2): 3 rows of maize intercropped with 2 rows of alfalfa, MA (3:3): 3 rows of maize intercropped with 3 rows of alfalfa. LSD; least significant difference, *** (*p* ≤ 0.001), ** (*p* ≤ 0.01).

## Data Availability

The original contributions presented in this study are included in the article/Supplementary Material. Further inquiries can be directed to the corresponding author .

## Supplementary Materials

The following supporting information can be downloaded at: https://www.mdpi.com/article/10.3390/plants14243846/s1, Table S1. Plot Size for Maize–Alfalfa Intercropping and Mono-cropping.
