# Supplementary material for: Optimizing Row Ratio Configurations for Enhanced Productivity and Resource-Use Efficiency in Maize–Alfalfa Intercropping
Source: Plants (Basel). 2025 Dec 17;14(24):3846. doi: 10.3390/plants14243846 (PMC12736538; doi:10.3390/plants14243846)
Supplement: Supplementary file 1 [file plants-14-03846-s001.zip › plants-4031428-supplementary.pdf]

Table S1. Plot Size for Maize–Alfalfa Intercropping and Mono-cropping

Treatment	Row Ratio (M:A)	Total Rows	Plot Width (m)	Plot Length (m)	Plot Area (m^2)
Intercrop 1	1:1	8	4.8	5	24
Intercrop 2	2:1	9	5.4	5	27
Intercrop 3	2:2	8	4.8	5	24
Intercrop 4	3:1	8	4.8	5	24
Intercrop 5	3:2	10	6.0	5	30
Intercrop 6	3:3	12	7.2	5	36
Maize mono-cropping	–	8	4.8	5	24
Alfalfa Mono-cropping	–	8	4.8	5	24
